# Statin-Associated Necrotizing Autoimmune Myositis Complicated by an Uncommon Adverse Effect to Treatment

**DOI:** 10.1155/2019/4601304

**Published:** 2019-06-25

**Authors:** Brian M. Fung, Emil R. Heinze, Andrew L. Wong

**Affiliations:** ^1^UCLA-Olive View Internal Medicine Residency Program, Sylmar, CA, USA; ^2^Division of Rheumatology, Department of Medicine, Olive View-UCLA Medical Center, Sylmar, CA, USA

## Abstract

Statin-associated necrotizing autoimmune myositis (NAM) is an autoimmune condition characterized by severe acute-onset proximal muscle weakness, a very high creatinine kinase (CK) level, and prominent myofiber necrosis and minimal lymphocytic infiltration on muscle biopsy. Unlike self-limited statin myopathy, this condition usually requires aggressive immunomodulation therapy to assist recovery and prevent future disability. In this case report, we present a patient who developed progressive muscle weakness after taking atorvastatin for one year. At initial presentation, her CK level was 28,000 U/L. She was diagnosed with statin-associated NAM and started on high-dose intravenous solumedrol, mycophenolate, and intravenous immunoglobulin (IVIG) therapy. However, she subsequently developed acute bilateral vision loss and right side hemineglect; she was diagnosed with posterior reversible encephalopathy syndrome (PRES), thought to be a possible delayed adverse reaction to IVIG. IVIG was discontinued, and the patient was treated with supportive therapy. At six-month follow-up, she had significant improvement in muscle strength and vision.

## 1. Introduction

Statin-associated myopathy has historically been thought of as a self-limited entity associated with statin use. However, over the past decade, an autoimmune variety of statin-associated myopathy has been recognized, with different characteristics from the self-limited disease; this immune-mediated entity was initially called statin-induced immune-mediated necrotizing myopathy (IMNM) and now commonly referred to as statin-associated necrotizing autoimmune myositis (NAM) [1]. This type of myopathy usually requires aggressive immunosuppression or immunomodulation therapy with corticosteroids and/or intravenous immunoglobulin (IVIG) therapy [2, 3]. Although IVIG is generally well tolerated and has been shown to contribute to high recovery rates [4], it is not without risks [5]. In this case report, we present a patient who developed posterior reversible encephalopathy syndrome (PRES), thought to be a possible delayed adverse reaction to receiving IVIG for treatment of statin-associated NAM.

## 2. Case Presentation

A 53-year-old woman with past medical history of type 2 diabetes mellitus, hyperlipidemia, and depression presented to the emergency department with progressive bilateral weakness over 6 months. She reported weakness that began in her lower extremities and then progressed to her upper extremities, affecting primarily her proximal muscle strength. She had no associated numbness or tingling, fevers, chills, headache, rashes or skin changes, joint pain, or recent injury. Her medications included metformin, glyburide, aspirin, and sertraline. She was also on a high-intensity statin for the past year without any recent dosage changes.

**Figure 1:**
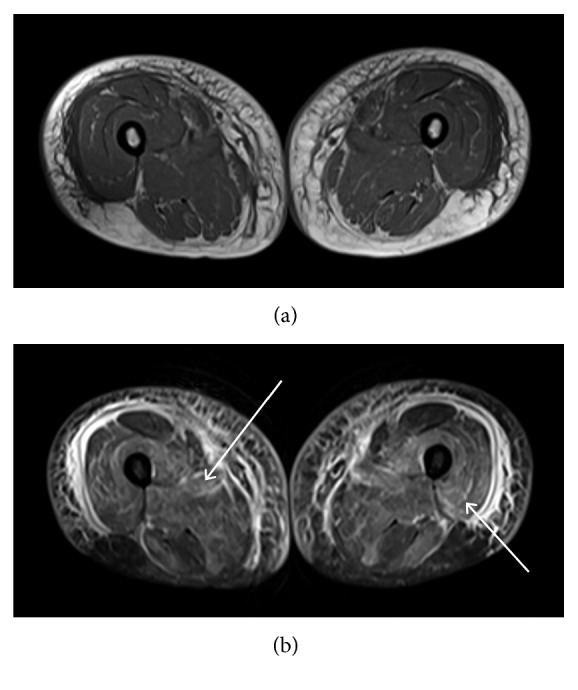
(a) T1-weighted and (b) short tau inversion recovery (STIR) sequences showing edema (hyperintense areas on STIR, white arrows) in the proximal thigh muscles, characteristic of an inflammatory myositis.

**Figure 2:**
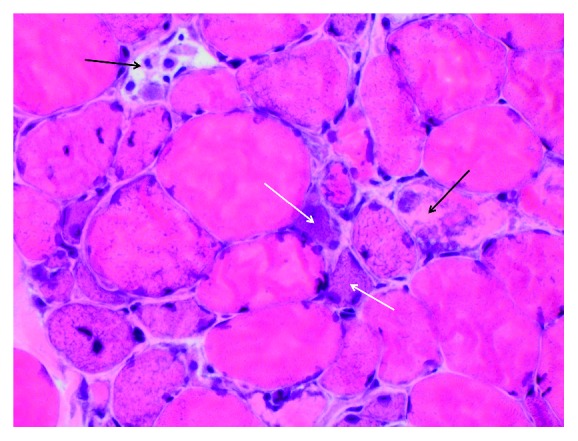
Hematoxylin and eosin-stained frozen section (400x magnification) of the vastus lateralis revealing marked fiber size variation with necrotic (black arrows) and regenerating (white arrows) myofibers consistent with a necrotizing autoimmune myositis.

Physical examination was significant for reduced muscle strength involving the neck, bilateral deltoids, and quadriceps. She appeared unsteady on her feet with a slightly widened gait. Deep tendon reflexes, sensation, and coordination were intact throughout all extremities. Initial labs were significant for a leukocytosis of 12,500 K/cumm, aspartate aminotransferase (AST) of 773 U/L, alanine transferase (ALT) of 763 U/L, erythrocyte sedimentation rate (ESR) of 35 mm/hr, C-reactive protein of 24 mg/L, and markedly elevated creatinine kinase (CK) of 28,000 U/L. ANA was 1 : 80 titer with a nucleolar pattern by HEp-2 indirect immunofluorescence (IF), and the anti-dsDNA antibody was negative by the *Crithidia luciliae* IF test (CLIFT). Magnetic resonance imaging (MRI) of the patient's pelvis revealed extensive edema throughout the proximal pelvic musculature with a symmetric distribution consistent with myositis (Figure 1). Furthermore, an electromyogram and nerve conduction study demonstrated diffuse and active irritable myopathy, and a muscle biopsy of the vastus lateralis revealed necrotizing myopathy with minimal inflammatory infiltrate and MHC1 immunostaining consistent with NAM (Figure 2).

Given the aforementioned findings, the patient was started on high-dose intravenous solumedrol, mycophenolate mofetil, and four consecutive days of IVIG for treatment of a necrotizing myositis (NM), which resulted in improvement in the creatinine kinase down to 8,000 after a week into therapy. An extended myositis panel and 3-hydroxy-3-methylglutaryl coenzyme-A (also known as HMG-CoA reductase or HMGCR) antibody test later resulted with positive PM/Scl-100 antibody (by qualitative immunoblot, ARUP Laboratories) and significantly elevated HMGCR antibody level (>200 units, by semiquantitative enzyme-linked immunosorbent assay, ARUP Laboratories), consistent with statin-associated NAM.

**Figure 3:**
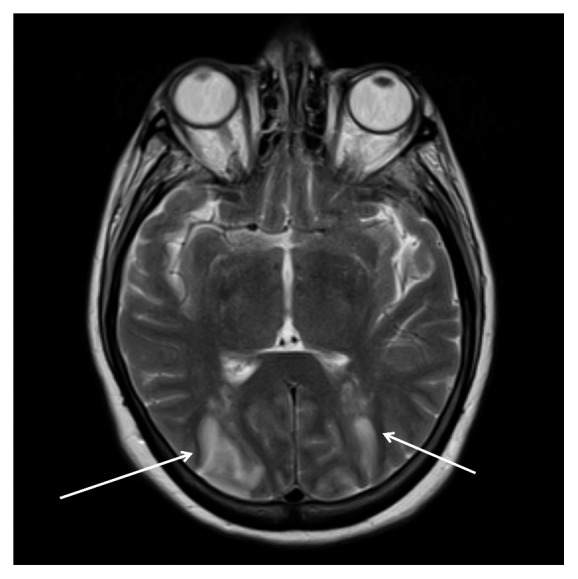
MRI T2-weighted sequence with hyperintense signal involving the occipital (arrows) and parietal lobes bilaterally, suggestive of posterior reversible encephalopathy syndrome.

About one week into the patient's treatment course, the patient developed acute bilateral vision loss and right side hemineglect. A magnetic resonance angiogram (MRA) of the head revealed development of diffuse arterial narrowing and irregularity consistent with cerebral vasospasm. Furthermore, she had areas of signal abnormality in the bilateral frontal, parietal, and occipital lobes with diffusion restriction. Consultation with neuroradiology suggested that the patient's neurological findings were consistent with PRES (Figure 3), suspected to be related to a delayed reaction to IVIG therapy. The patient was subsequently started on nimodipine and magnesium. Subsequent serial MRAs and neurological exams revealed radiographic and clinical improvement, respectively. However, her vision only improved minimally at that time. She was discharged with daily mycophenolate and sent to a rehabilitation facility to continue muscle strengthening and ambulation gait training. At 6-month follow-up, she reported marked improvement in physical strength and her vision was significantly improved; her CK returned to normal levels.

## 3. Discussion

Based on the patient's serological, histological, and clinical findings, a diagnosis of statin-associated NAM was made (anti-HMGCR-positive subset). Although the patient had a positive PM/Scl-100 antibody and an ANA with nucleolar pattern, she did not have any extra-muscular involvement such as interstitial lung disease, inflammatory joint disease, mechanic's hands, sclerodactyly, or Raynaud's phenomenon which would typically be seen in an overlap myositis (OM), such as an OM with scleroderma. However, it remains unknown whether she will develop additional symptoms over time. Patients with an idiopathic inflammatory myopathy (IIM) or autoimmune inflammatory myositis (AIM) can now more routinely be classified by their autoantibody pattern associated with different disease characteristics and treatment responses [1, 4]; however, we have not been able to find any studies of statin-associated NAM with a patient having both anti-HMGCR and anti-PM/Scl-100 antibodies simultaneously at the time of this report.

Statin-associated NAM is an autoimmune muscle disease (and subtype of IIM) characterized by prominent myofiber necrosis and minimal lymphocytic infiltration [6]. It is strongly associated with statin exposure and the development of HMG-CoA reductase antibody, although it can also occur in patients who have never taken a statin [6]. Compared to a self-limited statin myopathy, statin-associated NAM is more commonly associated with clinical proximal muscle weakness, higher creatinine kinase values, HLA-DRB1^*∗*^11:01 positivity, an irritable myopathy on EMG, diffuse muscle edema seen on MRI, and muscle necrosis with minimal inflammation on muscle biopsy [6–8]. It is important to note that time of onset is variable and may occur even years after statin exposure [6]. Simply discontinuing statin treatment in NAM is often inadequate as muscle damage and necrosis often continues even after cessation of the statin [6, 9]. Thus, most patients require aggressive immunosuppression or immunomodulation therapy, with first-line therapy including the use of high-dose corticosteroids and/or IVIG, as well as other immunotherapies such as methotrexate, azathioprine, mycophenolate, and/or rituximab, depending on the individual patient [2, 3, 6]. Interestingly, age appears to play a role in response to therapy, with a recent cohort study finding younger patients to have more severe disease and a worse prognosis compared to older patients [10]. Furthermore, it appears that earlier and more intense treatment is associated with improved outcomes [4, 7]. In this case, the patient was treated with a combination of corticosteroids, IVIG, and mycophenolate given her younger age and severe disease presentation. A recent study has found that human anti-HMGCR antibodies can induce muscle weakness in mice and appear to be directly pathogenic towards muscle through a complement-mediated mechanism; thus, in the future, plasma exchanges and complement-targeting therapies may also play a role in the treatment of NAM [11].

Although no randomized clinical trials have been performed to guide therapy of statin-associated NAM, IVIG has been shown to be a relatively safe and effective therapy for this autoimmune condition [4, 12]. Common adverse reactions include malaise, headache, and abdominal pain, although these reactions are generally mild [5]. However, IVIG has also been shown to be associated with several more serious adverse effects, including anaphylaxis, transfusion-associated lung injury, and thromboembolic events [13]; there are also a few case reports of PRES in patients receiving IVIG for neurological diseases such as Guillain–Barré and Miller–Fisher syndrome [14–17], including a case involving amelioration of PRES after IVIG treated early on with plasma exchange/immunoadsorption therapy [18]. However, from our review of the literature, there have been no published cases of PRES in a patient receiving IVIG for statin-associated NAM.

In this case, the patient's initial symptom of PRES was bilateral vision loss. Although the patient had a history of diabetes, a dilated fundus exam did not show any retinopathy, retinal ischemia, or anterior optic nerve involvement. Intraocular pressures were within normal ranges, and bilateral corneas and lens appeared normal. Furthermore, an MRI of the orbits was unremarkable, with normal appearing optic nerves and no intraconal mass identified. A systemic vasculitis related to the patient's newly diagnosed inflammatory myositis was also considered in the differential of the patient's neuroradiographic findings; however, the patient had interval progression of hyperintense lesions prior to improving (which would not be expected to be seen while on corticosteroid therapy). Thus, given the normal orbital and ocular structures, as well as the abnormal intracranial imaging findings, a diagnosis of PRES was made. In addition, the patient's clinical course suggests that IVIG may have been associated with the patient's development of PRES. The patient developed hyperintense lesions of her bilateral occipital regions and irregularities of the vertebral vessels after receiving IVIG treatment, similar to previously reported cases of PRES after administration of IVIG (although the reported timing of symptom onset in the literature is typically sooner, ranging between 24 hours after initiation of IVIG and 4–7 days after completion of IVIG therapy) [15–18]. Furthermore, she did not have any hypertensive episodes (or acute blood pressure fluctuations), kidney disease, signs or symptoms of infection, or electrolyte abnormalities that could otherwise explain the development of PRES [19–21].

PRES is a syndrome defined by neurological signs (most commonly headache, vomiting, and visual disturbances) and radiographic abnormalities (typically hyperintense signals on T2-weighted MR imaging especially in bilateral occipital regions, responsible for vision loss) [14]. Although little is known about the pathophysiology behind this disease process, it has been postulated that sudden changes in plasma viscosity induced by IVIG infusion, vasogenic edema, and cerebral vasospasm may lead to the development of PRES [14, 22]. Treatment of PRES involves cessation of the offending agent (in this case, the course of IVIG was already completed over one week before onset of symptoms) and strict blood pressure control when elevated [23]. Magnesium (often low in patients with PRES) should be repleted given its (prophylactic) anticonvulsive and vasodilating effects [24]. Furthermore, calcium antagonists are sometimes needed in the setting of cerebral vasospasm, as was the case in our patient [23, 25]. Improvement in neurological signs and symptoms is variable, depending on the initial severity of imaging and types of complications (e.g., progression of vasogenic edema to cytotoxic edema and ischemia) [26].

## 4. Conclusion

This clinical case report describes two suspected medication-induced adverse effects (statin-associated NAM and IVIG-induced PRES) in a single patient. We hope this report will serve as an important reminder that every medication can potentially have adverse effects (common, uncommon, and atypical), that the risks and benefits of each medication treatment must be considered, and that unusual/atypical adverse effects of even critical therapeutic medication treatments need to be recognized early, in order to optimize patient care outcomes.
